# 

**Published:** 1881-01

**Authors:** 


### CONTRIBUTORS.

| | |
|---|---|
| Professor H. C. Allen, | Ann Arbor. |
| " T. F. Allen, | New York City. |
| Doctor Edward Bayard, | " " " |
| Professor J. B. Bell, | Boston. |
| Doctor E. W. Berridge, | London. |
| " Ad. Fellger, | Phila. |
| " W. H. Jenny, | Kansas City, Mo. |
| " Ad. Lippe. | Phila. |
| " C. Lippe, | New York City. |
| " M. MacFarlan, | Phila. |
| " Thos. Moore, | " |
| " C. F. Nichols, | Boston. |
| " C. Pearson, | Washington. |
| " Thomas Skinner, | Liverpool. |
| " C. Swan, | New York. |
| " P. P. Wells, | Brooklyn. |
| " Wm. Wesselhoeft, | Boston. |
| Professor T. P. Wilson, | Ann Arbor. |
| &c. &c. &c. | |

N. B. Authors of accepted papers are furnished with copies of the number in which their articles appear; application for such copies to be made when sending papers.

